# Need for Dental Prostheses According to Obesity Levels in a Rural Population: A Cross-Sectional Study

**DOI:** 10.1155/ijod/9485502

**Published:** 2025-04-07

**Authors:** Javier Flores-Fraile, Sergio Parra-García, David Ribas-Pérez, Alejandro Moreno-Barrera, Luis El Khoury-Moreno, Diego Rodríguez-Menacho, Julio Torrejón-Martínez, Antonio Castaño-Seiquer, Juan Gómez-Salgado

**Affiliations:** ^1^Department of Surgery, Faculty of Medicine, University of Salamanca, Salamanca 37007, Spain; ^2^Department of Stomatology, Faculty of Odontology, University of Sevilla, Sevilla 41009, Spain; ^3^Department of Sociology, Social Work and Public Health, Faculty of Labour Sciences, University of Huelva, Huelva 21007, Spain; ^4^Safety and Health Postgraduate Programme, Universidad Espíritu Santo, Guayaquil 092301, Ecuador

**Keywords:** dental prostheses, diet, Mexico, obesity, oral epidemiology, oral health, prevention of periodontal disease, preventive dentistry, tooth wear, Yucatan

## Abstract

**Background:** Oral health is a crucial aspect of overall well-being, and its relationship with obesity has gained increasing attention due to the rising prevalence of obesity worldwide. Obesity is associated with various health complications, including detrimental effects on oral health. Despite the significant implications of obesity on dental health, there is limited research specifically examining this relationship in rural populations.

**Objective:** This study aimed to explore the relationship between obesity type and the need for dental prostheses in adults from the rural population of Yucatán,Mexico, in order to highlight the influence that obesity may have on oral health.

**Methodology:** An observational, descriptive, cross-sectional study was conducted in July 2021, with a final sample of 114 participants aged between 15 and 81 years. Subjects classified as obese according to the body mass index (BMI) criteria were included, specifically those with a BMI ≥ 30. All participants were evaluated by a single experienced dentist, using a standardized methodology to collect data regarding their oral health, clinical history, and health habits.

**Results:** A notable absence of dental prostheses was observed in over 80% (92 participants) of the subjects. A direct correlation was identified between obesity type and the need for prostheses, with a higher prevalence of prosthetic need in individuals with a higher BMI, where 75% of the obese (38 out of 50 obese participants) reported needing dental prostheses. This suggested a significant interplay between oral health, obesity, and dietary choices.

**Conclusions:** The findings of this study emphasized the importance of optimal oral health to facilitate chewing and digestion, highlighting that obesity, as a condition, can negatively influence oral health. Further studies are needed to investigate the necessity for preventive measures and treatment, as well as to promote awareness of oral health within the community.

## 1. Introduction

Oral health is a continuous state determined not only by hygiene and dietary habits but also by the absence of oral and systemic pathologies [1, 2]. Oral diseases currently exhibit high prevalence and incidence rates, directly impacting individuals' quality of life and affecting their daily performance due to discomfort such as pain, inflammation, and functional impairment [3–6].

More than 70% of the population suffers from some form of oral pathology, which contrasts with an ideal state of oral health characterized by complete dentition, absence of diseases, and proper dental relationships [6, 7]. In Mexico, the prevalence of oral diseases exceeds 90%, representing not only a local problem but also a significant sociohealth challenge [8–11]. Specifically in the Yucatán region, epidemiological data indicate rates of oral diseases comparable to those of the rest of the country, justifying the need to focus research efforts in this area.

Obesity, in turn, is a growing condition associated with numerous health issues, including oral health. Sociocultural and dietary factors contribute to the increasing incidence of obesity, particularly among younger populations, despite efforts to promote healthy lifestyles. This condition affects oral health by influencing chewing, bolus formation, and swallowing, which are crucial for digestion [12–14]. Previous studies have indicated a correlation between bite strength, which relates to chewing competence, and the need for dental prostheses in obese individuals. However, research specifically exploring this relationship in rural Mexican populations is scarce.

Few studies have specifically examined the interplay between obesity and oral health in the Mexican population. Although the consequences of obesity on overall health have been documented, the literature lacks investigations addressing how this condition relates specifically to the need for dental prostheses across different types of obesity. Therefore, it is crucial to fill this knowledge gap and contribute to understanding how obesity can affect oral health in the population of Yucatán.

The objective of this study is to relate the need and presence of dental prostheses in obese individuals to the type of obesity in a Mexican population in the state of Yucatán, highlighting the importance of addressing oral health in the context of obesity to promote improved quality of life.

## 2. Methods

### 2.1. Type of Study and Characteristics

An observational, descriptive, and cross-sectional study was carried out, covering the rural population of Merida (Mexico) and surrounding areas in July 2021.

This study was conducted in strict accordance with the Declaration of Helsinki and was approved by the Ethics Committee of the Fundación Odontología Social (Sevilla, Spain) under protocol code 01/2020, with approval granted on May 5, 2020. This information has been incorporated into the manuscript to ensure clarity and compliance with ethical requirements.

The study adhered to the STROBE (Strengthening the Reporting of Observational Studies in Epidemiology) checklist. The initial sample size was 170 patients. These patients attended a routine check-up at their public health center without prior knowledge that they would be part of a study to avoid biases. The sampling method was therefore nonprobabilistic and incidental.

#### 2.1.1. Inclusion Criteria

Age: Include subjects aged 12 years or older, as the research focuses on adults and the relationship between obesity and the need for dental prostheses. This automatically exclude minors who may still have mixed or incomplete dentition.

Dentition: Include only those with complete or partially lost permanent dentition, who no longer have any deciduous teeth, but may still require prostheses. It is more relevant to focus on individuals with current or potential prosthetic needs.

General health status: Include only participants who are in a condition to be evaluated odontologically and who do not present severe systemic diseases that could affect the study's outcome (e.g., uncontrolled metabolic disorders and active cancer treatment).

Obesity: Include only participants with complete and recent data on weight, height, and body mass index (BMI) to accurately classify the level of obesity, which is a key variable in the study.

#### 2.1.2. Exclusion Criteria

Deciduous or mixed dentition: Specifically exclude patients with temporary or mixed dentition, that is, individuals under 12 years of age or adults who still have deciduous teeth.

Recent dental treatment: Exclude patients who have received prosthetic dental treatment in the last 6 months, as they may have a lower immediate need for prostheses, potentially biasing the results.

Extreme oral health issues: Exclude individuals with severe periodontal diseases or oral pathologies that impede an adequate assessment of prosthetic need, as these conditions may influence the need for treatment differently.

Uncontrolled systemic diseases: Exclude subjects with uncontrolled severe systemic diseases (e.g., uncontrolled diabetes and severe hypertension) that may confound or distort the effects on both obesity and oral health.

After the application of these criteria, the final sample size was 114 patients over 12 years who met the criterion of having permanent dentition. Of these, 70% of the initial sample were women and this percentage was 67.5% in the final sample. All were informed of the process orally and the explanation was extensive and personalized. They all signed an informed consent form and also received an information sheet wrote in plain language. After this, they filled out a research dossier with questions about their oral and dental health, anamnesis and clinical history, well-balanced health habits according to an ideal model of Mediterranean diet, body measurements, and socioeconomic level in addition, but it has no further value in this investigation due to the main purpose.

All patients had the same clinical examiner, ensuring that all patients were evaluated consistently and uniformly, without variations in the data collection procedure to avoid bias. It was decided to carry out the scanning for data collection through the work from a single examiner with a significant level of expertise and clinical competence, particularly in assessing the need for dental prostheses. With the aim to measure the consistency of the observations, the examiner was subjected to a so-called intraobserver calibration, obtaining the ratio of agreement with a Kappa test (0.85). Also, patients were unaware that they would be participating in the study until they arrived at the health center, which helped to avoid selection bias.

### 2.2. Variables

The study variables were gender, degree of obesity, use of prosthesis, and need for prosthesis. The central variable of the study was the degree of obesity, which was in turn divided into three degrees according to BMI, these being:

1. Type I: BMI from 30 to 34.99
2. Type II: BMI from 35 to 39.99
3. Type III: BMI greater than 40.00

The use of prosthesis is understood as the presence of fixed or removable, denture-supported, implant-supported, dentomucosal-supported, or mucosal-supported prosthesis present in the mouth at the time of the examination.

The need for prosthesis is understood as the clinical demand or the requirement according to the criteria of evidence-based dentistry, for fixed or removable prosthesis, denture-supported, implant-supported, dentomucosal-supported,or mucosal-supported, whether demanded or not, and whether in combination with other types of prosthesis at the time of the examination. The need for prosthesis does not include the demand for purely esthetic criteria that do not result in an improvement in masticatory function or an improvement in oral and oral-facial quality of life, including at the articular level.

These study variables were obtained by routine and voluntary examination of the patients who came to receive dental care at the “International Resident Dentist by University of Salamanca” campaign site. The documentary completion of the data was carried out by the same explorer, as well as its transcription into a validated research dossier. All methods employed were rigorously selected based on well-established scientific standards in epidemiological and dental research. The procedures for data collection, anthropometric measurements, and dental assessments followed validated and standardized protocols.

### 2.3. Statistical Analysis

The Kolmogorov–Smirnov test was performed to evaluate the normality of the distribution of the variables. For data that did not follow a normal distribution, the Kruskal–Wallis test and the ANOVA test were used for data with normal distribution in descriptive statistics.

Fisher's exact test was used for the comparison of tables between two groups and the chi-square test for contingency tables of all groups. A multivariate analysis was performed to determine the relationship between the dependent and independent variables, using multiple regression analysis.

The analysis was performed using the automatic statistical calculator IBM SPSS Statistics for Windows, Version 25.0. Statistical significance was accepted for *p*  < 0.05.

## 3. Results

### 3.1. Age

The mean age was 39.38 years, with a SD ± 15.70. A median of 37.50 years was obtained and the range of ages seen was from 15 to 81 years old.

**Figure 1:**
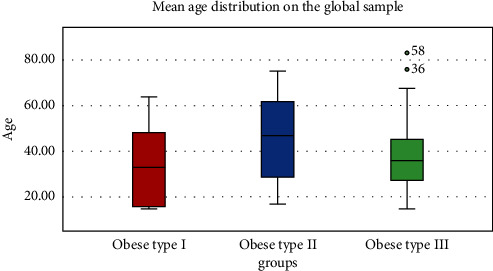
Mean age in the obesity groups (Oral Health in Yucatan, Mexico, 2020–2023).

Table 1 and Figure 1 show the age in the obesity groups. There were no significant differences (*p*=0.177).

### 3.2. Sex

Table 2. shows sex distribution per type of obesity in the general population studied.

There was no significant difference between the sex of the population and the degrees of obesity.

The group with the highest percentage of women was in the type III obesity group with 68.7% and the highest percentage of men was in the type I obesity group with 44.4% with a *p*-value of 0.725 (nonstatistical significance). There is a higher percentage of women 67.5% than men 32.5% within the general sample.

**Figure 2:**
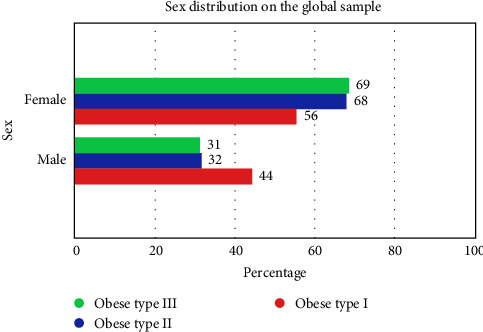
Sex distribution in the overall sample (Oral Health in Yucatan, Mexico, 2020–2023).

Figure 2 shows the sex distribution in the overall sample with no statistical significance.

### 3.3. Use of Dental Prosthesis

Table 3 shows the distribution of the use of dental prostheses by group in the overall sample.

**Figure 3:**
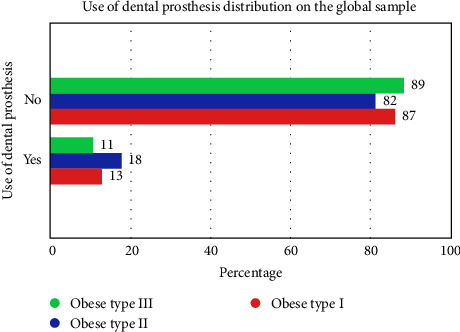
Distribution of the use of dental prostheses in the overall sample (Oral Health in Yucatan, Mexico, 2020–2023).

There are no significant differences between the use of dental prostheses in the population and the groups. The highest percentage of use of dental prosthesis was in obese type II with 18.2% and the highest percentage without use of dental prosthesis was in obese type I with 88.9%. There was a higher percentage of no denture use with 73.5% compared to denture use with 68.8%.  Figure 3 shows the distribution of the use of dental prostheses in the overall sample.

### 3.4. Needs for Dental Prosthesis

Table 4 shows the distribution of the need for dental prostheses by groups in the general sample.

There were no significant differences between the need of dental prostheses in the population and the groups.

**Figure 4:**
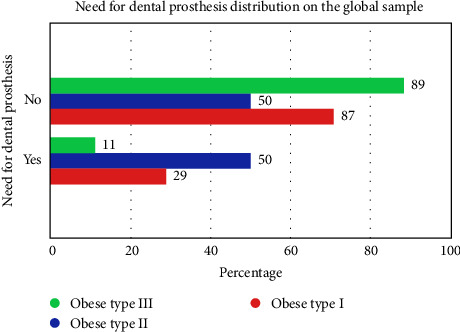
Distribution of the need for dental prostheses in the overall sample (Oral Health in Yucatan, Mexico, 2020–2023).

The highest percentage of need of dental prostheses was in obese type II with 50% and the highest percentage without need of dental prostheses was in obese type I with 88.9%. There was a higher percentage of no need of prosthesis with 75.6% compared to the need of prosthesis with 66.7%.  Figure 4 shows the distribution of the need for dental prostheses in the general sample.

## 4. Discussion

Obesity is defined as an excessive or anomalous deposit of adipose tissue in the body, which has harmful consequences for health, according to Vara [15]. This is caused by an excess of calories ingested by the diet with respect to the basal calories consumed by the organism according to E. Culebras-Atienza, F. J. Silvestre, and J. Silvestre-Rangil [16].

The oral cavity of a healthy adult without agenesia presents 32 teeth in normal conditions, showing in this way and in case of an optimal disposition of the same, a maximum efficiency in the development of the masticatory process and formation of the alimentary bolus, indispensable as initial part of the digestion [17].

The Mexican population has a high rate of obesity, which is a serious socioeconomic problem for the country that has been addressed from different perspectives. This problem has been attributed to cultural and lifestyle factors. However, it has not been possible to delve into the origin of the situation and the reasons for this lifestyle [9–11]. According to the results obtained after collecting information from 170 people between the ages of 15 and 81 years, some alarming numbers are reflected, while the distribution by gender is slightly tending towards the female gender with 60%, nonuse of dental prostheses in the sample is 86% on average. Meanwhile, 31.6% of the total sample need dental prostheses, which means that some people need prostheses despite not having it.

Women in the three groups of obesity are the ones who present a higher proportion, in addition (55.6%, 68.2%, and 68.7% in each group from types I to III with *p*-value of 0.725), as their size within the total sample is greater, we can make a hypothesis according to the higher proportion in sample that they are the ones who have less use of prosthesis and greater need. This may be due to several reasons: on the one hand, in Mexico, the birth culture is oriented towards large families and several styles confirm the presence of altered oral health conditions during pregnancy, many of them involving tooth loss (pregnancy caries). Secondly, Mexican society is an eminently traditional society (especially in the lower and middle social classes), so that the jobs with the greatest physical performance are performed by men. These jobs involve a higher caloric expenditure that can compensate to some extent for the excessive caloric intake.

With regard to the use of prostheses, as mentioned above, 86% of the subjects in the sample did not have prostheses, even though they needed them in a global proportion of 31.6%. This happens not only because of the economic factor but also because of the lack of need since they adapt their lifestyle to function with a chewing that is inefficient. This also conditions the fact of not being able to ingest foods with hard or fibrous consistency due to the impossibility or discomfort.

Due to this, the intake of processed foods of soft consistency, sugary drinks, and other types of refined products characterized by their easy swallowing and softening in contact with saliva increases. It is observed that all groups of obesity are characterized notably by the nonuse of dental prostheses as far as 86%, however, the total need of dental prosthesis is 31.6%. The prevalence of use and no need of prosthesis is higher in types I and III obesity with an inversely proportional relationship with no significant differences according to *p*-values.

The use of prostheses in turn corelates inescapably with the need to wear prostheses, whether removable or fixed. This is important because if there is no need to wear a prosthesis, the concept of prosthesis use would become irrelevant since optimal masticatory efficiency would be assumed. However, in the case of the Yucatecan population under study, this is not the case. It is observed that the higher the type of obesity and therefore the higher the BMI, the greater the need to wear prostheses. It is curious how there is a significant difference between type I obesity with a demand of 11% and type III with a demand of 29%. The correlation between the type of obesity and the need for prostheses is therefore direct. This can confirm the hypothesis that the greater the need for prostheses, the lower the intake of healthy foods due to the impossibility of optimal chewing and crushing for integration into the food bolus.

After all the above, we must suppose a direct relationship between obesity and the need for prostheses, an inverse relationship between obesity and the use of prostheses and with a greater tendency for the female gender due to a higher proportion in our sample (*H*₀: there is no significant relationship between obesity and the need for dental prostheses in the adult population of Yucatán). This hypothesis must be confirmed with deep studies over this certain field and differentiating the fact that a person can use prosthesis and need prosthesis because of the degeneration of his oral condition and prevalence by gender and age, for example. However, this association, although it seems evident, is not strong enough in this research, which is confirmed by others such as that of Aceves-Martins et al. [18] in which the correlation between oral health and obesity in Mexican adolescents is studied, and it is obtained that although oral health is apparently related to obesity, it is not only important to address this from the dental point of view but also from the point of view of vital habits and the promotion of a healthy lifestyle [19]. This is confirmed in another study also by Aceves-Martins et al. [18] where it is concluded that the sociocultural factors of Mexican society are largely responsible for the high prevalence of obesity and are the root of the problem that must be eradicated.

However, this contrasts with the research of Shin et al. [20] which concludes in their article that poor oral health and poor mastication resulting from the absence of one or more teeth and the need for prostheses are not only statistically related to obesity, but especially to early cognitive impairment. This study was developed in a South Korean population in 2019 and confirms the importance of good oral health resulting in optimal food intake and swallowing.

In contrast, P. Gaewkhiew, W. Sabbah, and E. Bernabé [21] found no statistically significant association between the absence of functional dentition and overweight, but did find an association between the presence of complete functional dentition and normal and underweight. All this was studied in a Thai population. Regarding the number of teeth, a study by Singh et al. [22] in a Brazilian population concluded that there is no direct relationship with statistical significance between the number of missing teeth and central obesity, which contrasts with the research carried out here. However, what is concordant is that women are more prone to obesity and even more so when they have some tooth loss.

Therefore, although they did not find an association between dental loss and obesity at a general level, they do give importance to the fact of gender in the face of the probability of being obese. In this case being a woman, which is consistent with the data collected in the Mexican population and which can be extrapolated from one study to the other due to geographical proximity and demographic resemblance. All of the above, however, contrasts with the line also carried out in the Brazilian population by Peruchi et al. [23] which in its article concludes that there is a direct and statistically significant relationship between poor oral hygiene and tooth loss, with obesity. Moreover, she adds that poor oral hygiene, even when wearing a removable prosthesis to rehabilitate edentulism, also predisposes you to be obese. Therefore, not only is the absence of teeth related to obesity, but even if they are present with removable prostheses if accompanied by poor hygiene, it is also a risk factor.

Also, in favor of the hypothesis to be demonstrated here is the study by Yamazaki et al. [24] in which they investigated whether having a mandibular overdenture improves food intake and nutritional status in edentulous adults compared with a conventional complete denture and with edentulous people who have not been rehabilitated. The conclusion they reach is that there is no direct association between this difference in rehabilitation (complete denture vs. mandibular overdenture) beyond patient comfort, but there is a difference with respect to edentulous adults, as a priori shown by the data of the study concerned.

Despite all the above, this research can provide a basis on which to carry out studies on the importance of optimal oral health with the presence of all the dental roots in the first phases of digestion and formation of the alimentary bolus. This oral health allows an optimal performance in the ingestion of medium/high consistency foods, many of which are associated with the gold dietary standard that is the Mediterranean diet, which are vegetables, fruits, nuts, meat, and bread, etc.. It is advisable to continue carrying out studies that provide more evidence in this regard and that create a basis for social awareness of the importance not only of treatment but also of prevention in terms of oral health.

About the limitations of our study, a number of constraints should be considered when interpreting our findings. The study presented is part of a Social Dentistry solidarity project and given the heterogeneity of the sample, it does not allow us to make an initial estimate for carrying out larger studies in the future. The design is also a limitation because it does not allow us to set causality relationship. Also, the sampling method should be considered as a limitation, because randomization was not performed.

In this sense, although our sample included 114 participants, its size may not be fully representative of the broader rural population of Yucatán. This limitation affects the generalizability of our results and suggests the need for further studies with larger, more diverse samples.

Second, the inclusion and exclusion criteria may have introduced additional variability. By encompassing a wide age range (2–81 years), the study captured a broad spectrum of oral health conditions and prosthetic needs. However, this heterogeneity may not have been fully accounted for in the analysis, potentially influencing the interpretation of results.

Third, the cross-sectional design of our study limits the ability to establish causal relationships between obesity and the need for dental prostheses. While our findings suggest an association, longitudinal studies are necessary to explore causality and better understand the temporal dynamics of this relationship.

Fourth, data collection relied on self-reported information regarding health habits and clinical history. This approach, while practical in community-based studies, introduces the possibility of recall bias or self-reporting bias, which could affect the accuracy of the collected data.

Finally, our study did not include data on additional factors such as socioeconomic status, educational background, or access to dental care—variables that could significantly influence both oral health and obesity. Future research should incorporate these elements to provide a more comprehensive understanding of the observed associations.

Also, this study may have the bias of studying a sample of Yucatan, which cannot be related to the whole population of Mexico, due to the huge differences among states of the country. Focusing on our sample, investigators can reduce bias by increasing the number of people to represent Yucatan population at least, but COVID-19 restrict the mobility of people who wanted to come up to the health centers at that time. It must be considered to restudy this hypothesis with the aim of supporting scientific associations among degrees of obesity, use of dental prosthesis, need of dental prosthesis, and gender mainly. Despite these limitations, our study provides valuable insights into an understudied population, highlighting the need for further research to expand upon and refine these findings.

The main conclusion of the study is the suggestion that the need for prostheses in Mexican adults shows a hypothetical direct association with degrees of obesity. Maybe, it can be related also to female gender, supposing more frequent that the absence of prosthesis uses and the greater need for it occurs in adult women with type III obesity. More studies are needed in order to focus on this presumptive relationship and other risk factors should be studied to confirm this association with more evidence or increase the knowledge of interaction between degrees of obesity and presence or not of a complete dentition., focusing on variables like gender, age, diet, or type of oral prosthesis.

## Figures and Tables

**Table 1:** Age in the obesity groups (Oral Health in Yucatan, Mexico, 2020–2023).

Group	Mean	SD	Median	Range
Type I	34.88	18.46	33.00	15–64
Type II	45.40	18.50	47.00	17–75
Type III	38.27	14.34	36.00	15–81

**Table 2:** Sex distribution by groups in the overall sample (Oral Health in Yucatan, Mexico, 2020–2023).

Cross table between sex and study groups						
			Groups			Total
			Obese type I	Obese type II	Obese type III	
Sex	Female	Count	5	15	57	77
		Within the sex (%)	6.5%	19.5%	74.0%	100.0%
		Within the groups (%)	55.6%	68.2%	68.7%	67.5%
		Total (%)	4.4%	13.2%	50.0%	67.5%
		Corrected residual	−0.8	0.1	0.4	—
	Male	Count	4	7	26	37
		Within the sex (%)	10.8%	18.9%	70.3%	100.0%
		Within the groups (%)	44.4%	31.8%	31.3%	32.5%
		Total (%)	3.5%	6.1%	22.8%	32.5%
		Corrected residual	0.8	−0.1	−0.4	—

*Note:* Chi-square: 0.643, *p*-value: 0.725.

**Table 3:** Distribution of the use of dental prostheses by group in the general sample (Oral Health in Yucatan, Mexico, 2020–2023).

Cross table between study groups and use of dental prostheses						
			Group use of dental prostheses			Total
			Obese type I	Obese type II	Obese type III	
Use of dental prostheses	No	Count	8	18	72	98
		Within no use of dental prostheses (%)	8.2%	18.4%	73.5%	100.0 %
		Within group (%)	88.9%	81.8%	86.7%	86.0 %
		Corrected residual	0.3	−0.6	0.4	—
	Yes	Count	1	4	11	16
		Within use of dental prostheses (%)	6.3%	25.0%	68.8%	100.0 %
		Within group (%)	11.1%	18.2%	13.3%	14.0 %
		Corrected residual	−0.3	0.6	−0.4	—

*Note:* Chi-square: 0.419, *p*-value: 0.811.

**Table 4:** Distribution of the need for dental prostheses by groups in the general sample (Oral Health in Yucatan, Mexico, 2020–2023).

Study groups and need for dental prosthesis						
			Group need for dental prosthesis			Total
			Obese type I	Obese type II	Obese type III	
Need for dental prosthesis	No	Count	8	11	59	78
		Within need for dental prosthesis (%)	10.3%	14.1%	75.6%	100.0%
		Within group need for dental prosthesis (%)	88.9%	50.0%	71.1%	68.4%
		Corrected residual	1.4	−2.1	1.0	—
	Yes	Count	1	11	24	36
		Within need for dental prosthesis (%)	2.8%	30.6%	66.7%	100.0%
		Within group need for dental prosthesis (%)	11.1%	50.0%	28.9%	31.6%
		Corrected residual	−1.4	2.1	−1.0	—

Total		Count	9	22	83	114
		Within need for dental prosthesis (%)	7.9%	19.3%	72.8%	100.0%
		Within group need for dental prosthesis (%)	100.0%	100.0%	100.0%	100.0%

*Note:* Chi-square: 5.473, *p*-value: 0.065.

## Data Availability

The data will be made available upon request from the authors.
